# Rapid Grading of Fundus Photographs for Diabetic Retinopathy Using Crowdsourcing

**DOI:** 10.2196/jmir.3807

**Published:** 2014-10-30

**Authors:** Christopher J Brady, Andrea C Villanti, Jennifer L Pearson, Thomas R Kirchner, Omesh P Gupta, Chirag P Shah

**Affiliations:** ^1^Wills Eye HospitalRetina Service: Mid Atlantic RetinaPhiladelphia, PAUnited States; ^2^Wilmer Eye InstituteJohns Hopkins University School of MedicineBaltimore, MDUnited States; ^3^Schroeder Institute for Tobacco Research and Policy StudiesLegacyWashington, DCUnited States; ^4^Ophthalmic Consultants of BostonBoston, MAUnited States

**Keywords:** diabetic retinopathy, telemedicine, fundus photography, crowdsourcing, Amazon Mechanical Turk

## Abstract

**Background:**

Screening for diabetic retinopathy is both effective and cost-effective, but rates of screening compliance remain suboptimal. As screening improves, new methods to deal with screening data may help reduce the human resource needs. Crowdsourcing has been used in many contexts to harness distributed human intelligence for the completion of small tasks including image categorization.

**Objective:**

Our goal was to develop and validate a novel method for fundus photograph grading.

**Methods:**

An interface for fundus photo classification was developed for the Amazon Mechanical Turk crowdsourcing platform. We posted 19 expert-graded images for grading by Turkers, with 10 repetitions per photo for an initial proof-of-concept (Phase I). Turkers were paid US $0.10 per image. In Phase II, one prototypical image from each of the four grading categories received 500 unique Turker interpretations. Fifty draws of 1-50 Turkers were then used to estimate the variance in accuracy derived from randomly drawn samples of increasing crowd size to determine the minimum number of Turkers needed to produce valid results. In Phase III, the interface was modified to attempt to improve Turker grading.

**Results:**

Across 230 grading instances in the normal versus abnormal arm of Phase I, 187 images (81.3%) were correctly classified by Turkers. Average time to grade each image was 25 seconds, including time to review training images. With the addition of grading categories, time to grade each image increased and percentage of images graded correctly decreased. In Phase II, area under the curve (AUC) of the receiver-operator characteristic (ROC) indicated that sensitivity and specificity were maximized after 7 graders for ratings of normal versus abnormal (AUC=0.98) but was significantly reduced (AUC=0.63) when Turkers were asked to specify the level of severity. With improvements to the interface in Phase III, correctly classified images by the mean Turker grade in four-category grading increased to a maximum of 52.6% (10/19 images) from 26.3% (5/19 images). Throughout all trials, 100% sensitivity for normal versus abnormal was maintained.

**Conclusions:**

With minimal training, the Amazon Mechanical Turk workforce can rapidly and correctly categorize fundus photos of diabetic patients as normal or abnormal, though further refinement of the methodology is needed to improve Turker ratings of the degree of retinopathy. Images were interpreted for a total cost of US $1.10 per eye. Crowdsourcing may offer a novel and inexpensive means to reduce the skilled grader burden and increase screening for diabetic retinopathy.

## Introduction

Since early diabetic retinopathy (DR) is often asymptomatic, detection of disease at this stage is either incidental or by deliberate screening. Screening for DR is both effective and cost-effective [1-4], but adherence rates to published guidelines for screening for DR are low, with only 35-60% of diabetic patients receiving an annual dilated fundus examination in the United States [5-8]. As a way to increase adherence, telehealth screening using non-mydriatic fundus photography and remote interpretation is increasing, especially in rural and remote settings [9-11]. Early diagnosis of DR and institution of appropriate therapy represents an enormous opportunity to prevent vision loss in a young, working-age demographic [3,4]. Telehealth, in particular, may be a way to control provider, payer, and societal costs.

Among the costs of a telehealth program are the fundus camera, the telehealth software package, and the human resources needed for image acquisition and interpretation. Fundus photo interpretation costs in diabetic retinopathy screening may be high given the labor-intensive interpretation protocols and the need to interpret multiple images per patient. Computerized, semi-automated image analysis techniques have been developed that may be able to reduce physician workload and screening costs [12-14]; however, these methods are not FDA-approved, nor in wide use clinically at this time. If telehealth continues to expand, low-cost methods will be needed to interpret the large volume of fundus images expected with rising incidence of diabetes, especially in resource-poor settings and in large public health screenings.

Crowdsourcing is defined by Brabham as “an online, distributed problem-solving and production model that leverages the collective intelligence of online communities to serve specific organizational goals” [15]. A subset of crowdsourcing, which he terms “distributed-human-intelligence tasking”, can involve subdividing larger tasks into small portions and then recruiting a group of individuals to each complete these small portions, and only collectively, the entire task [15]. The use of crowdsourcing in biomedical research is in its infancy, though some groups have used this method in public health research [16] and to interpret medical imaging. For example, malaria researchers have used a Web-based game to recruit untrained, anonymous volunteers to tag and count malaria parasites on digital images of blood smears [17]. The investigators showed that by combining the analyses of several users, they were able to achieve similar accuracy rates to expert microscopists. Crowdsourcing has recently been used to categorize a number of fundus photos with a variety of diagnoses as normal or abnormal [18]. In a trial conducted in the United Kingdom using untrained graders, the sensitivity was ≥96% for normal versus severely abnormal and between 61-79% for normal versus mildly abnormal [18].

The current research uses diabetic retinopathy as the test condition to explore whether a crowdsourcing interface can be used to train workers to classify human fundus photos as normal or abnormal and subsequently conduct diagnostic grading of images [19]. This project estimates the validity and reliability of crowdsourced grading of images for diabetic retinopathy, compared to the criterion standard of expert grading. Our hypothesis is that crowdsourced grading of fundus photography interpretation can be rapid, accurate, and reliable in the screening for diabetic retinopathy.

## Methods

### Crowdsourcing

**Figure 1:**
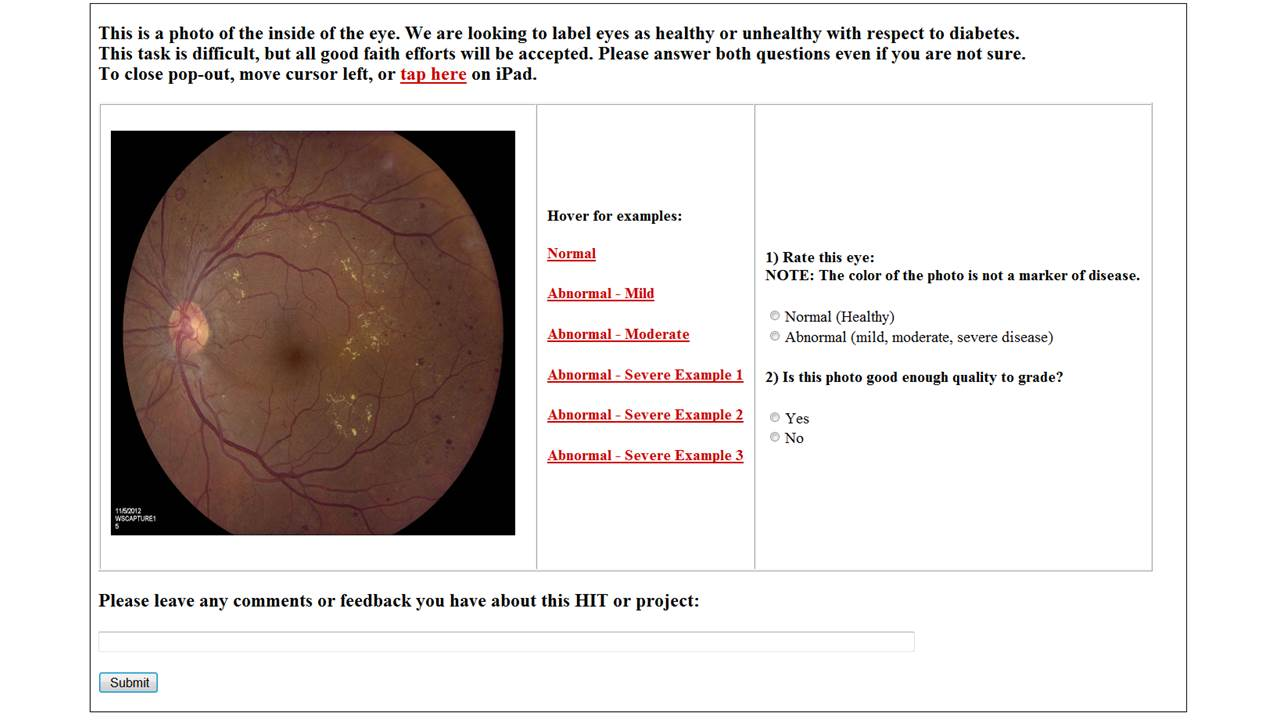
Screenshot of the Amazon Mechanical Turk Web interface for fundus photo grading.

An interface for fundus photo classification was developed for the Amazon Mechanical Turk (AMT [20]) crowdsourcing platform (Figure 1). AMT is an online labor market that allows access to thousands of people who can quickly accomplish small, discrete tasks for small amounts of money. Typical AMT tasks include tagging photos, translating words, or writing very short articles for websites. AMT has also been used to annotate photos of the tobacco point-of-sale retail environment [21], evaluate oral health promotion materials [22], investigate the relationship between depression and stigma [23], assess people’s reactions to frightening anti-smoking campaigns [24], and evaluate public awareness of ovarian cancer [25], among many other research-orientated applications [26-28]. Amazon Mechanical Turk has its own vocabulary used by AMT workers (Turkers) and AMT task administrators (Requestors). A Human Intelligence Task (HIT) is a small job that may be performed in a matter of seconds or minutes and, once the work is approved by the requestor, may pay US $0.01-$0.25 or more per task depending on the complexity of the HIT. A group of HITs is called a “batch” and is made up of similar HITs. Depending on the complexity of the task and the payment offered by the Requestor, a batch is often completed within minutes or hours of posting.

AMT is a reputation-based economy such that Turkers may only access the most desirable HITs once they have a sufficient track record of previously accepted work [29]. High quality Turkers may avoid a new Requestor’s HITs until the Requestor has demonstrated his or her own fairness in approving and rejecting work. Indeed, a Turker’s reputation will suffer following rejection of even a small number of HITs. AMT is a complex ecosystem in which both high-quality work on the part of the Turkers and fairness on the part of the Requestor are rewarded.

Turkers perform their work anonymously, but demographic studies have been conducted. In a survey of 1000 Turkers, Ipeirotis found that 46.8% of Turkers are located in the United States, 34% are in India, and the remaining 19.2% were from 64 other countries [30]. The majority of workers in the United States were women, most of whom reported AMT as a source of supplemental income, whereas in the majority of workers in India were men, and reported AMT as their primary source of income. Across nations, Turkers were younger and better educated than the general population [30].

### Design

For the current study, the United Kingdom national screening program grading scale [31] was used by 2 attending retinal surgeons (OPG, CPS) to categorize 19 anonymized teaching images. The same AMT interface and resolution of images used by the Turkers was used by the expert graders. Consensus was reached by discussion on images for which there was disagreement. For the purposes of the study, terms from the UK grading scale were translated into plain language: “background” retinopathy was called “mild”, “preproliferative” was called “moderate”, and “proliferative” was called “severe”. “Maculopathy” was defined as abnormal on a training image with otherwise moderate disease but was not coded separately. The AMT interface was designed to provide training on grading of DR within each HIT. This training included 6 images annotated with the salient features of each level of retinopathy in plain language. Turkers were presented with the following text: “This is a photo of the inside of the eye. We are looking to label eyes as healthy or unhealthy with respect to diabetes. Rate this eye.” Turkers could hover their mouse over 6 adjacent training images (1 normal, 1 mild, 1 moderate, 3 severe) while reviewing the active test image. This layout allowed for all of the training and grading to occur in one browser window. More examples of proliferative/severe disease were provided due to the heterogeneity of findings constituting this level of disease. There were no restrictions on the type of device or display/monitor used by Turkers to complete the task.

### Phase I

In the first phase of the study, the 19 images were posted to AMT for grading by Turkers, with 10 repetitions per photo for an initial proof-of-concept. Four photos were re-posted with this initial batch for 10 repetitions to assess intragrader reliability. Turkers were paid US $0.10 per image, and a 10% commission was paid to Amazon. In order to be eligible to view and complete the HITs, Turkers needed to have successfully completed 100 prior HITs and have an overall HIT approval rate of 97%.

In the initial batch, HITs were posted asking Turkers to grade images as normal (0) versus abnormal (1). In subsequent batches, Turkers were asked to grade the same 19 images using three categories (normal=0, mild to moderate=1, and severe=2) and then four categories (normal=0, mild=1, moderate=2, and severe=3). Percentage of images correctly classified was calculated. Sensitivity and specificity were calculated for all batches, collapsing all abnormal categories together for three- and four-category grading tasks using Stata 13. A worker consensus grade was assigned to each image based on the mode of the 10 Turker scores. Consensus grade using the mean of the 10 Turker scores was also calculated on an exploratory basis. For the two category tasks, a mean score <0.50 was defined as normal, and ≥0.50 was abnormal. For the three category tasks, <0.50 was defined as normal, ≥0.5 to <1.5 was defined as mild to moderate, and ≥1.5 was defined as severe. In the four category tasks, <0.50 was defined as normal, ≥0.5 to <1.5 was defined as mild, ≥1.5 to <2.5 was defined as moderate, and ≥2.5 was defined as severe.

### Phase II

The purpose of Phase II was two-fold. First, Phase II sought to evaluate the emergent ability of the crowd to accurately distinguish between different levels of retinopathy, based on the idea that larger numbers of raters would increasingly coalesce around the correct answer. Second, Phase II sought to identify and ultimately confirm the threshold beyond which the contribution of one more rater would cease to provide additional information. To accomplish this, one prototypical image from each of the four grading categories was submitted to undergo 500 unique Turker interpretations. Jackknife resampling methods were then used to draw random subsamples from this “population” of 500 Turkers, beginning with 50 random samples of 2 Turkers, then 50 random samples of 3 Turkers, and so forth [21,32]. This made it possible to estimate the variance in accuracy derived from each randomly drawn sample of raters at each crowd size and to compute area under the curve (AUC) of the receiver-operator characteristic (ROC) curve, indicating their performance relative to the expert grading.

### Phase III

In Phase III of the study, three additional iterative batches of the 19 images were run in an effort to improve Turker grading using the four diagnostic categories. The first batch used Turker feedback from all previous batches to modify the training image set. The second batch used more stringent criteria for Turkers in addition to the modified training images. A minimum of 500 completed and approved HITs was required as was an overall HIT approval rate of 99%. The third batch was conducted using Turkers holding an Amazon designation of “Photo Moderation Master” and raised the compensation to US $0.15 per image, in addition to the changes made for batches 1 and 2. The criteria necessary to achieve this designation are not published, but Masters are “elite groups of Workers who have demonstrated accuracy on specific types of HITs on the Mechanical Turk marketplace. Workers achieve a Masters distinction by consistently completing HITs of a certain type with a high degree of accuracy across a variety of Requesters” [33]. As in Phase I, percent correctly classified, worker consensus score, and average time to complete the HITs were estimated for these three iterative batches.

The Wills Eye Institute Institutional Review Board ruled that approval was not required for this study.

## Results

### Phase I

Two expert graders (OPG, CPS) coded 12 images as abnormal and 7 as normal (Table 1). Each of the three Phase I batches consisted of 23 photos (19 unique, 4 duplicates) with 10 unique graders for a total of 230 grading instances. Time-to-complete Turker grading of images varied with the number of grading categories. Two category (normal/abnormal) grading was completed in 20 minutes, three-category grading in 3 hours, and four-category grading in 2 hours. Because the images were interpreted rapidly and workers could complete as many or as few of the HITs as desired, most of the four duplicate images were rated by unique Turkers and therefore, we were unable to assess intragrader reliability.

Across 230 grading instances of unique images in the two-category HITs (normal vs. abnormal) of Phase I, 187 (81.3%) of the images were correctly classified by Turkers (Table 1). Sensitivity and specificity were 93.6% and 67.8% respectively using individual Turker scores. Sensitivity and specificity were 100% and 71.4% respectively using Turker consensus scores. Average time to grade each image was 25 seconds, including time to review training images. At US $0.11 per grading, each image was graded for $1.10, and grading garnered an effective hourly wage of $14.31 (Table 2).

Overall number of correctly classified images decreased with the addition of a third and fourth grading category to 64.4% (148/230) and 50.9% (117/230), respectively. Specificity and specificity for individual Turkers was 96.3% and 66.7% respectively for both three and four categories. At the level of Turker consensus, sensitivity was 100% for both three and four categories, and specificity was 71.4% and 100% for three and four categories, respectively. With additional grading criteria, the speed of grading decreased, as did the effective hourly wage. Average time to complete the three-category HITs was 51 seconds, for an effective hourly wage of $7.08. Average time to complete the four-category HITs was 55 seconds, for an effective hourly wage of $6.60 (Table 2).

**Table 1:** Turker grading of individual images^a^.

Image #	Two-category rating			Three-category rating			Four-category rating		
	Expert rating	Correct diagnosis^b^, %	Turker consensus^c^	Expert rating	Correct diagnosis^b^, %	Turker consensus^c^	Expert rating	Correct diagnosis^b^, %	Turker consensus^c^
1	Nor	65	—	Nor	90	—	Nor	55	—
2	Ab	85	—	M/M	50	Sev	Mild	0	Sev
3	Nor	70	—	Nor	70	—	Nor	70	—
4	Nor	50	Ab	Nor	40	M/M	Nor	60	—
5	Nor	80	—	Nor	70	—	Nor	50	—
6	Ab	100	—	M/M	90	—	Mild	20	Mod
7	Ab	90	—	Severe	60	—	Sev	10	Mod
8	Nor	50	Ab	Sev	40	M/M	Nor	65	—
9	Ab	100	—	Sev	95	—	Sev	100	—
10	Ab	100	—	Sev	40	M/M	Sev	70	—
11	Ab	90	—	Sev	0	M/M	Sev	20	Mild
12	Nor	90	—	Nor	80	—	Nor	90	—
13	Ab	100	—	M/M	30	Sev	Mod	20	Sev
14	Ab	80	—	Sev	40	M/M	Sev	10	Mod
15	Nor	90	—	Nor	100	—	Nor	90	—
16	Ab	90	—	Sev	70	—	Sev	50	—
17	Ab	100	—	M/M	60	—	Mild	10	Mod
18	Ab	100	—	M/M	100	—	Mod	95	—
19	Ab	90	—	M/M	80	—	Mild	20	Mod
Correct, %		81.3	89.5		64.4	63.2		50.9	57.9
Sensitivity^d^, %		93.6	100.0		96.3	100.0		96.3	100.0
Specificity^d^, %		67.8	71.4		66.7	71.4		66.7	100.0

^a^Nor=Normal; Ab=Abnormal; M/M=Mild or Moderate; Sev=Severe; Mod=Moderate.

^b^At the level of the individual graders.

^c^Consensus rating presented only if it differed from the expert rating.

^d^Calculated for normal versus any disease level.

**Table 2:** Time to complete ratings (in seconds).

	Two-category rating	Three-category rating	Four-category rating	Four-category rating (improved training)	Four-category rating (increased approval)	Four-category rating (Master Graders)^a^
Mean time per HITs	25.16	50.87	54.52	50.98	38.79	44.14
95% CI	21.93-28.38	43.18-58.55	46.15-62.88	39.66-62.30	31.65-45.93	36.00-52.27
Hourly wage, $	14.31	7.08	6.60	7.06	9.28	12.23
Cost per image, $	1.10	1.10	1.10	1.10	1.10	1.95

^a^Master graders received US $0.15 per image, plus a 30% Amazon commission for a total cost of US $0.195/image.

### Phase II

**Figure 2:**
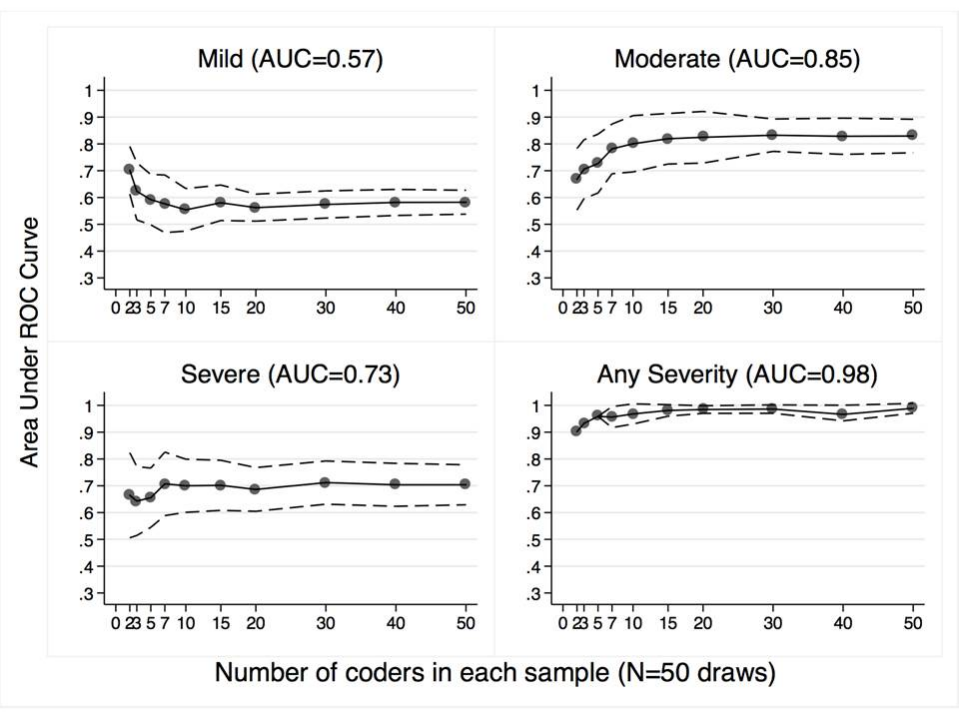
Area under the curve (AUC) of the receiver-operator characteristic (ROC) curve for increasing numbers of Turker interpretations of a prototypical image from each severity level. Turkers had low accuracy for the Mild (Panel A) and Severe image (Panel C), but acceptable accuracy for the Moderate image (Panel B). When all four images were analyzed for absence or presence of disease only, Turkers performed well (Panel D) with a highly significant AUC.

Results of Phase II likewise indicate that sensitivity and specificity for overall ratings of abnormal versus normal was excellent, producing a highly significant AUC (0.98; Figure 2, Panel D). Turkers were not as accurate when asked to differentiate among four severity levels. Post hoc contrast analyses, however, indicate that Turkers performed well when asked to identify the abnormalities that were moderate in severity (ROC=0.85; Figure 2, Panel B). The pattern of results indicates that lower accuracy identifying mild (ROC=0.57; Figure 2, Panel A) and severe (AUC=0.73; Figure 2, Panel C) abnormalities was due to a tendency to rate all abnormalities as moderate in severity, rather than a failure to recognize normal versus mild and severe abnormalities more generally. Results also indicate that maximum AUC was usually achieved when crowd size reached a total of between 7 and 10 Turkers, confirming the validity of the crowd sizes used to rate the larger set of images (Figure 2). This affirms that the results of Phases I and III would not have been different had we sought a larger number of Turkers for each HIT.

### Phase III

In Phase III, an additional normal training image was added due to Turkers’ interpreting visible choroidal vessels as abnormal during Phase I. The annotations were clarified to indicate that the presence of a single microaneurysm is considered abnormal and that hard exudates need not be present to achieve moderate or severe disease. Functionality to enlarge the image being graded was added. In the first batch using the new training, the percentage of correctly classified images using the consensus Turker scores was no better than previous: 42.1% (8/19 images) versus 57.9% (11/19 images), respectively by mode and 21.1% (4/19 images) versus 26.3% (5/19 images) by mean. In the second batch, with more stringent criteria for Turker selection, the proportion of correctly graded images improved to 52.6% (10/19 images) by mean (mode unchanged). This result was confirmed in a repeat run of this exact batch (data not shown). In the third batch, using “Photo Moderation Masters”, the proportion of correctly graded images decreased to 36.8% (7/19 images) by mean (mode unchanged). Throughout all batches, the diagnostic sensitivity for any level of disease was maintained at 100% (Table 3)

**Table 3:** Turker consensus in Phase III.

	Number correct (mean)^a^	% correct (mean)	Number correct (mode)^a^	% correct (mode)	Sensitivity^b^	Specificity^b^
Phase I: Four-category rating	5	26.3	11	57.9	100.0	100.0
Phase 3: Trial 1 (improved training)	4	21.1^c^	8^d^	42.1	100.0	57.1
Phase 3: Trial 2 (raised approval rating)	10	52.6	11^e^	57.9	100.0	100.0
Phase 3: Trial 3 (Master Graders)	7	36.8	11	57.9	100.0	100.0

^a^Calculated by level (eg, Turker consensus matches expert designation as normal, mild, moderate, and severe).

^b^Calculated for normal versus any disease level using the mode consensus score.

^c^After excluding a single Turker with systematically higher scores, 42.1% correct.

^d^Three images had no mode and were considered incorrect for “Number Correct” and “% correct” but recoded as abnormal for sensitivity and specificity.

^e^One image had no mode and was considered incorrect for “Number Correct” and “% correct” but recoded as abnormal for sensitivity and specificity.

## Discussion

### Principal Findings

With minimal training, an anonymous, untrained workforce recruited through a public crowdsourcing platform can rapidly and correctly categorize fundus photos of diabetic patients as normal or abnormal. The Turkers in this study graded these images with a high sensitivity, which is ideal for a screening instrument. Critically, no false negative consensus results were generated in any of our batches, indicating that no cases of DR were missed. When asked to categorize photos by degree of retinopathy, Turkers improved with changes in the Turk interface, particularly with increasing prior approval rating needed to perform the HITs. The number of graders required to reach a reliable “consensus grade” was consistent across categories, and 10 grading instances per image was established as sufficient for valid results.

Images were interpreted for a total cost of US $1.10 per eye. While abnormal images would still currently still require further evaluation to identify patients in need of a live examination, this cost is near the limit suggested by some investigators for feasible public health screening in developing economies [34]. Indeed, the reimbursement per image, which ranged from an effective hourly wage of US $6.60 to $14.31 in our study, could possibly be reduced, since a reasonable target hourly wage for AMT workers is the federal minimum wage of US $7.25 per hour or even lower [35]. Additionally, posting larger batches of images might allow for lower per image reimbursement, since Turker speed would likely increase after becoming more skilled at the task, allowing them to maintain the same effective wage. While there may not be a direct relationship between quality responses and high wages [36], there may be a link between high wages and rapid completion of image grading, so it may not be wise to dramatically reduce reimbursement.

A post hoc analysis of individual Turkers’ scores revealed inconsistent use of the four grading categories by one Turker in the first batch of Phase 3 (Table 3). Several issues are brought to light by considering this specific batch. First, inconsistent use of all categories was a rare occurrence, demonstrating that Turkers are conscientious workers. This was also evident from comments made by Turkers as they completed HITs, which included thoughtful suggestions for improvement to the interface and concern over HITs that were felt to be ambiguous. Second, using the mean of crowdsourced responses may generate outputs that are rather sensitive to outliers. For this reason, using the mode to calculate consensus is generally preferable, though some images may not have a pure mode (Table 3), in which case the higher score of any “tie” would be used clinically. Third, and more broadly, AMT may be susceptible to Turker accounts that attempt to take advantage of the system by rapidly completing HITs with random responses either with live individuals or with automated programs or “bots” [29]. Moving forward, it may be necessary to analyze raw Turker scores for such phenomena and perhaps develop methods to exclude systematically unreliable scores.

Since AMT is a reputation-based economy, Requestors can reject or block Turkers who are not performing appropriately. Both actions negatively impact the Turkers’ reputation, which in turn affects their ability to perform HITs in the future, so there is a strong incentive to perform tasks accurately and honestly. This is likely why increasing the prior HIT approval rating to 99% had the most dramatic impact on consensus accuracy. Adding the “Photo Moderation Master” qualification did not improve worker consensus. This may be due to the fact that the criteria Amazon uses to grant this qualification are not relevant to our task. Additionally, since only a fraction of Turkers have the qualification, requiring it reduces the available workforce, which can increase the time required to complete batches. Especially when factoring in the additional Amazon commission, use of the Master qualification may not be necessary or cost-effective for these types of tasks in the future.

The current study was limited to a small set of hand-picked, high-quality, mydriatic fundus photos chosen to illustrate the key findings in diabetic retinopathy. Screening populations might have a subset of low-quality or uninterpretable images and would also be expected to have far more images of normal fundi. Identifying pathology in such sets would require extra vigilance on the part of Turkers to detect mild disease within large groups of normal photos. Larger datasets with more subtle pathology need to be tested with this methodology. Additionally, analyzing whether iterative improvements to the interface lead to better results is confounded by the fact that Turkers may have previously been exposed to the task and may be improving in their grading through practice. This is unlikely because the Turkers receive no feedback on their grading, so they do not know if they have correctly graded images or not. Moreover, while it is not currently feasible to “block” Turkers who have previously completed any of our HITs, it is possible to view their grading history within the project. Surprisingly, throughout all batches posted, most of our HITs were completed by Turkers otherwise naïve to our project. In the final batch of 190 HITs posted for this project, after approximately 3000 HITs using the same images had been posted, 170 (89.5%) were completed by Turkers who had never done any of our HITs before, and 20 HITs were done by 3 individual Turkers who had each graded only four images previously (data not shown). In future larger batches, adjusting for individual graders’ improvement over time could become necessary.

### Future Considerations

While further refinement of the methodology is still needed to resolve the degree of retinopathy, the current model could possibly be used as a way to reduce the burden on skilled graders by eliminating most of the normal images in very large screenings and passing only those abnormal images on for further characterization. While the individuals who complete HITs on AMT are truly anonymous, they do have unique numerical identifiers and can be tracked across HITs and batches of HITs. Therefore, an intriguing possibility using a crowdsourcing interface could include developing a cadre of specific Turkers who demonstrate a track record of reliable grading. These graders might be capable of a higher level of categorization than the general pool of Turkers and could be recruited for more complex grading tasks. Additionally, it is likely that automated computer algorithms will also play a role in the analysis of fundus images for DR and other conditions in the future. This raises the possibility of an even more robust interaction between artificial intelligence and human intelligence. Images could be graded in a tiered fashion by one system, and then those graded ambiguously could be routed to the other for further validation.

An unanticipated benefit of such a crowdsourcing program is that it might raise awareness of diabetes and diabetic retinopathy. Since our interface allowed Turkers to leave feedback for us to refine the instrument, we were able to capture comments such as, “I have learn about diabetes little bit [sic]”, “I really liked seeing the pics of the eye, very interesting”, and “This HIT was very good and a nice break from all of the bubbling surveys. Thank you!”, suggesting an interest in the subject matter beyond other HITs and beyond what we had expected at the outset. This finding is consistent with what has been termed “Virtual Citizen Science” in fields outside of biomedical research [37].

### Conclusions

Crowdsourcing represents a novel and inexpensive means to rapidly identify diabetic retinopathy. Further refinements of the technique are required, as is external validation with larger image sets. Additionally, multiple medico-legal and ethical issues would need to be addressed prior to clinical use of this technology, but there may be a role for crowdsourcing medical imaging data in large public health screenings and other settings in the future.

## Abbreviations

AMT: Amazon Mechanical Turk
AUC: area under the curve
DR: diabetic retinopathy
HIT: human intelligence task
ROC: receiver-operator characteristic
